# Field evaluation of the BG-Malaria trap for monitoring malaria vectors in rural Tanzanian villages

**DOI:** 10.1371/journal.pone.0205358

**Published:** 2018-10-08

**Authors:** Elis P. A. Batista, Halfan Ngowo, Mercy Opiyo, Gasper K. Shubis, Felician C. Meza, Doreen J. Siria, Alvaro E. Eiras, Fredros O. Okumu

**Affiliations:** 1 Laboratório de Ecologia Química de Insetos Vetores, Departamento de Parasitologia, Instituto de Ciências Biológicas, Universidade Federal de Minas Gerais, Belo Horizonte, Brazil; 2 Environmental Health and Ecological Sciences Department, Ifakara Health Institute, Ifakara, Tanzania; 3 School of Public Health, Faculty of Health Sciences, University of the Witwatersrand, Parktown, Republic of South Africa; 4 Institute of Biodiversity, Animal Health and Comparative Medicine, University of Glasgow, Glasgow, United Kingdom; Fred Hutchinson Cancer Research Center, UNITED STATES

## Abstract

BG-Malaria (BGM) trap is a simple adaptation of the widely-used BG-Sentinel trap (BGS). It is proven to be highly effective for trapping the Brazilian malaria vector, *Anopheles darlingi*, in field conditions, and the African vector, *Anopheles arabiensis*, under controlled semi-field environments, but has not been field-tested in Africa. Here, we validated the BGM for field sampling of malaria vectors in south-eastern Tanzania. Using a series of Latin-Square experiments conducted nightly (6pm-7am) in rural villages, we compared mosquito catches between BGM, BGS and human landing catches (HLC). We also compared BGMs baited with different attractants (Ifakara-blend, Mbita-blend, BG-Lure and CO_2_). Lastly, we tested BGMs baited with Ifakara-blend from three odour-dispensing methods (BG-Cartridge, BG-Sachet and Nylon strips). One-tenth of the field-collected female *Anopheles gambiae s*.*l*. and *Anopheles funestus* were dissected to assess parity. BGM captured more *An*. *gambiae s*.*l*. than BGS (p < 0.001), but HLC caught more than either trap (p < 0.001). However, BGM captured more *An*. *funestus* than HLC. Proportions of parous *An*. *gambiae s*.*l*. and *An*. *funestus* consistently exceeded 50%, with no significant difference between methods. While the dominant species caught by HLC was *An*. *gambiae s*.*l*. (56.0%), followed by *Culex* spp. (33.1%) and *Mansonia* spp. (6.0%), the BGM caught mostly *Culex* (81.6%), followed by *An*. *gambiae s*.*l*. (10.6%) and *Mansonia* (5.8%). The attractant-baited BGMs were all significantly superior to un-baited controls (p < 0.001), although no difference was found between the specific attractants. The BG-Sachet was the most efficient dispenser for capturing *An*. *gambiae s*.*l*. (14.5(2.75–42.50) mosquitoes/trap/night), followed by BG-Cartridge (7.5(1.75–26.25)). The BGM caught more mosquitoes than BGS in field-settings, but sampled similar species diversity and physiological states as BGS. The physiological states of malaria vectors caught in BGM and BGS were similar to those naturally attempting to bite humans (HLC). The BGM was most efficient when baited with Ifakara blend, dispensed from BG-Sachet. We conclude that though BGM traps have potential for field-sampling of host-seeking African malaria vectors with representative physiological states, both BGM and BGS predominantly caught more culicines than *Anopheles*, compared to HLC, which caught mostly *An*. *gambiae s*.*l*.

## Introduction

Between 2010 and 2015, there have been declines of more than 50% in malaria cases worldwide [1]. Intensification of vector control programs, such as use of insecticide treated bed nets (ITNs) and indoor residual spraying (IRS) is recognized as the major causes of this decline [2]. However, the World Health Organization (WHO) estimates that since 2016 the number of malaria cases is now increasing again [1] and in many low and middle-income endemic areas, such as different regions of Africa, Asia and South America, malaria and other mosquito-borne illnesses still remain major public health challenges [1, 3, 4]. Improved vector surveillance tools are needed to monitor epidemiological trends, measure impacts of current interventions, and identify areas requiring specific resources or interventions. Although difficult to achieve, such tools should be effective in different areas, so that the data obtained can be compared across regions. Besides, for these tools to be considered representative, they should capture similar species diversities and physiological states of mosquitoes as those that would normally bite humans.

In many residual transmission settings in Africa and South America, there is need to intensify measurements of transmission. In east and west Africa, widespread use of long-lasting insecticide treated bed nets (LLINs) and IRS has caused significant changes in the composition of malaria vector populations during the past decade [5–8]. Proportions of early-biting and outdoor-biting vectors have now risen, and as a result, available tools for monitoring the vectors are not sufficiently effective [9–11]. The human landing catch (HLC) remains the most effective method for collecting anophelines in these settings, although it is a dangerous procedure, which involves volunteers exposing their legs to potentially infectious mosquito bites, and may also be affected by inherent differences in attractiveness among the human volunteers [12]. Recent advances developed by Ifakara Health Institute and partners, such as the electric grid trap [13, 14] and the odour-baited mosquito landing boxes fitted with electric grids [15], appear to offer better and safer alternatives, but their application remains constrained by high costs, difficulties in battery power storage and risk of human contact with electric grid.

The BG-Sentinel trap (BGS) [16], developed by Biogents, Germany, is among the most widely used mosquito traps around the world. It is mostly used for surveillance of *Aedes* mosquitoes, but has also been tested for *Anopheles* mosquitoes [17–19]. Recently, the BG-Malaria trap (BGM) was conceived by adapting the BGS, with minor modification, to improve its efficacy for sampling *Anopheles* species, and was demonstrated as an efficacious method for monitoring malaria vectors in Brazil [20, 21]. The BGS and BGM traps differ in the airflow orientation, i.e., the BGM is set upside down, 40 cm above the ground [20–22]. This trap was demonstrated to be particularly effective for sampling the Brazilian malaria vector, *Anopheles darlingi*. In initial field tests conducted in Brazil, the BGM caught consistently more *An*. *darlingi* than the other commercial traps tested, including BGS and CDC light traps, and performed almost as well as HLC [20].

More recently in controlled semi-field trials conducted using laboratory-reared *Anopheles arabiensis* inside large screened-cages in Tanzania, the BGM was consistently better than BGS [22]. No such tests have however been conducted with wild free-flying malaria mosquitoes anywhere in Africa. Here, we evaluated the BGM for field sampling of malaria vectors in three rural malaria endemic villages in south-eastern Tanzania. This current study was therefore also meant to validate the efficacy of the BGM as previously shown in Brazilian field studies [20, 21] and the Tanzania semi-field studies [22].

## Methods

### Study area

**Fig 1:**
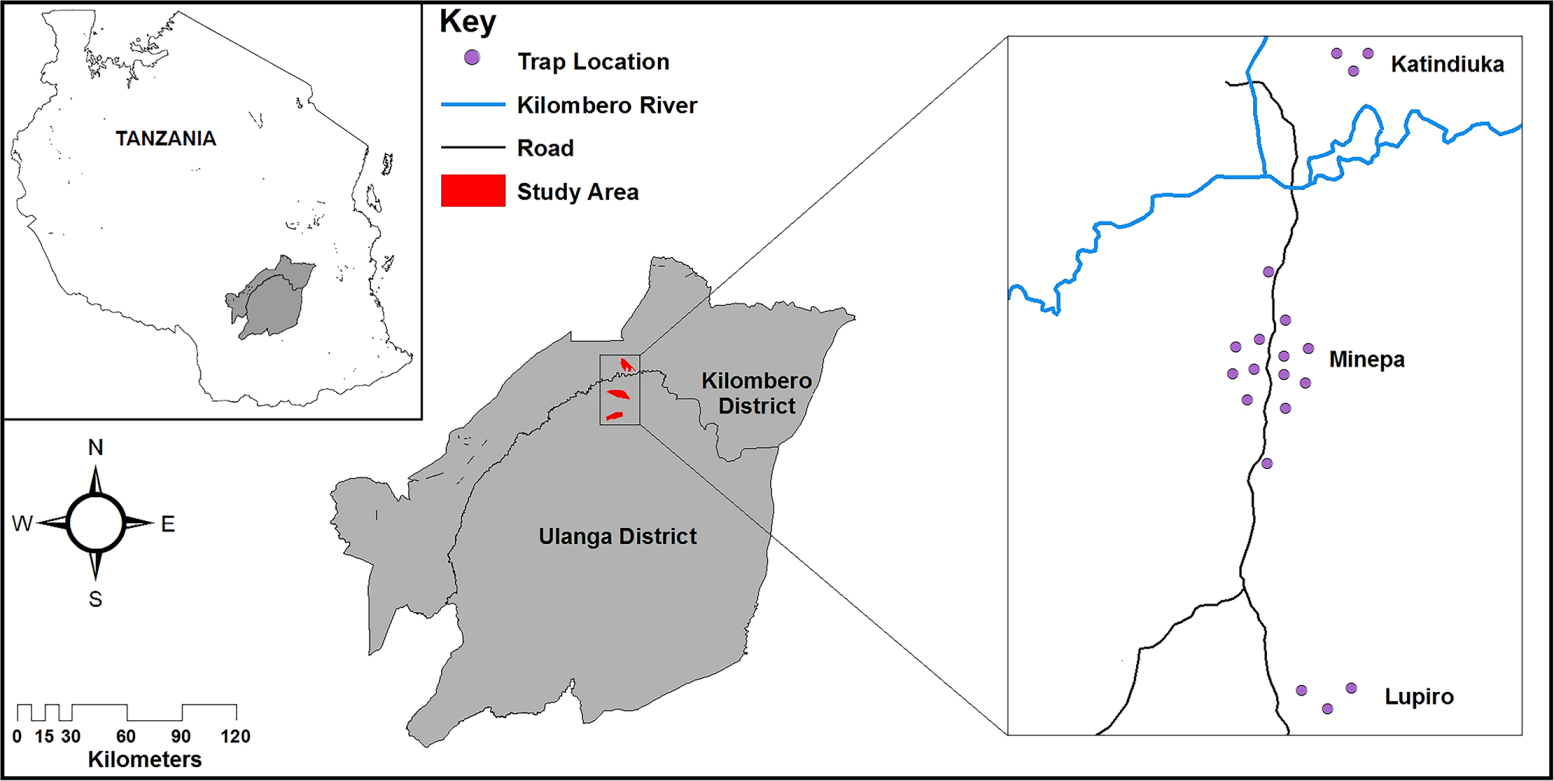
Map of the study area showing the trap positions (circles) in the villages in Ulanga and Kilombero districts in Tanzania, where the study was conducted.

The work was conducted in three villages in Ulanga (Katindiuka village) and Kilombero (Minepa and Lupiro villages) districts in rural south-eastern Tanzania (Fig 1). The daily mean temperature in the area ranges from 20°C to 32.6°C. The rainfall varies between 1200 to 1600mm, annually, with a main rainy season from March to May and a short rainy season during October to December. The other months of the year, January-February and June-September, correspond to the hot and cold dry seasons, respectively. This region is a perennially meso-endemic malaria area, where the vectors comprise primarily *Anopheles funestus* and *Anopheles gambiae s*.*l*. complex, of which *Anopheles arabiensis* constitute >99% of the complex [15, 23–25]. *Anopheles rivulorum* is also often incriminated with *Plasmodium* sporozoites, maybe an important secondary vector species in the area [26].

### Mosquito sampling methods

#### BG-Sentinel trap (BGS)

**Fig 2:**
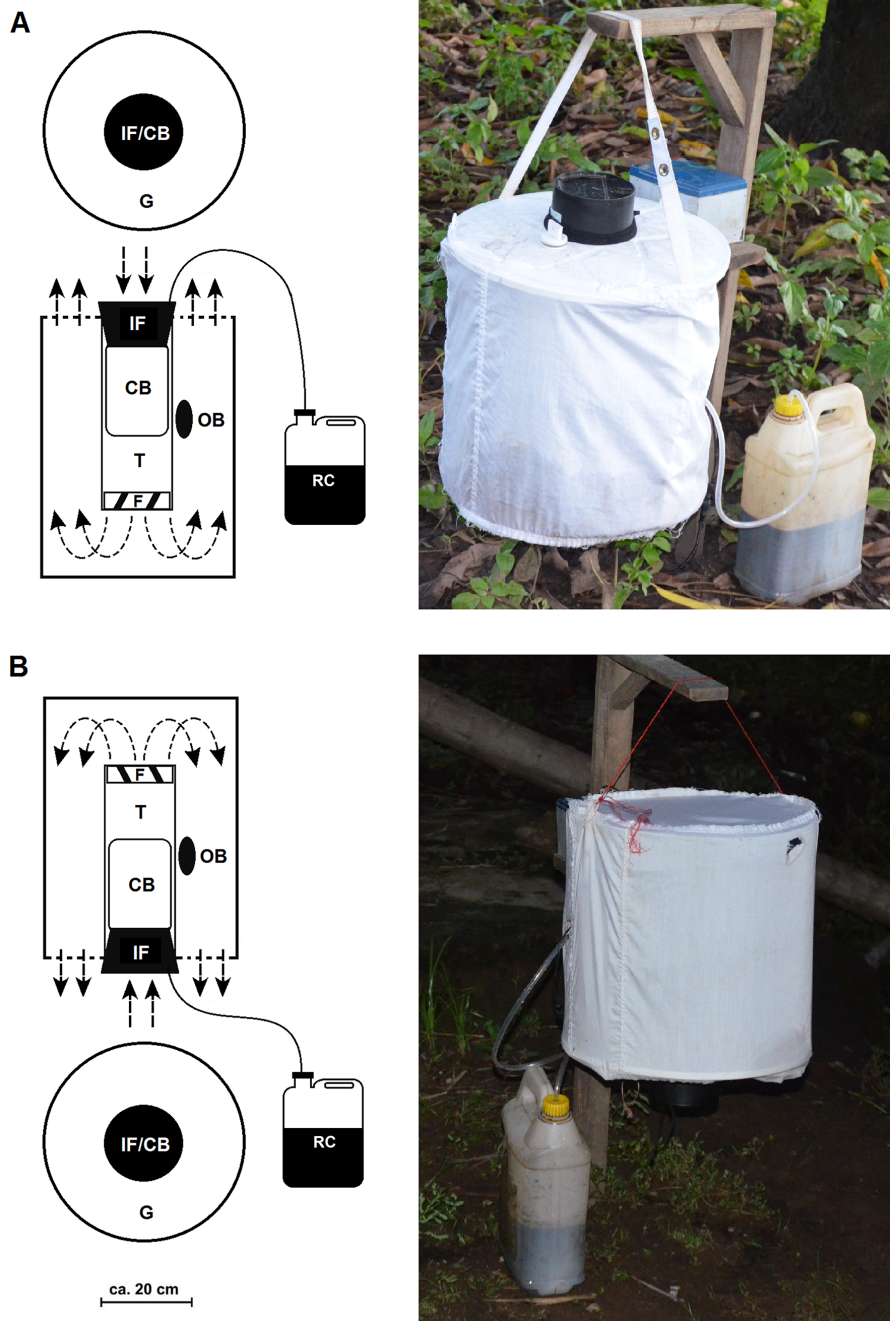
**Sampling methods: (A) BG-Sentinel and (B) BG-Malaria.** The functionality of the traps is shown on the left panel, while the installation in field is shown on the right panel of the figure. IF = Intake funnel; CB = Catch Bag; F = Fan; G = Gauze Cover; T = Tube; RC = Recipient of CO_2_; OB = Odour Bait. Arrows indicate the direction of the airflow. Adapted from Batista *et al*., [22].

The BGS (Biogents HmGb, Regensburg, Germany), shown in Fig 2, is a collapsible, waterproof fabric cylindrical container white trap, measuring 35cm in diameter and 40cm in height [16]. At the centre of the trap there is a black collecting tube of 12cm diameter and 30cm height) with a bag for the collection of mosquitoes. A 12 volt electric fan measuring 14cm in diameter and powered by 12 volt battery, produces a continuous downward flow of air, which hits the floor of the trap and exits through a gauze cover on the top of the trap. The downward air current produced by the fan draws in the mosquitoes that approach the collecting tube. The spacious interior allows for addition of a wide variety of attractants, odours which exit through the gauze cover and serve to lure mosquitoes to the collecting tube. The trap was installed outdoors with the entry at 40cm above the floor.

#### BG-Malaria trap (BGM)

The BGM is a simple adaptation of BGS trap and has been described in detail by both Gama *et al*. [20] and Batista *et al*. [22]. The BGM is hung upside down, at 40cm above the ground, and has an electrical fan (12V, 14cm diameter, powered by a 12 volt battery), which produces an upward suction that captures mosquitoes approaching the trap [20]. In the studies done by Gama *et al*. [20], the BGM was also tested with different black and white patterns to create a level of contrast essential for host seeking mosquitoes. However in this current set-up, the main difference between the two traps is the airflow orientation. The BGM was also installed outdoors with the entry at 40cm above the floor, as illustrated by Fig 2.

#### Human landing catch (HLC)

As a standard reference, human landing catches were conducted outdoors nightly by trained volunteers, with field experience in the collection of mosquitoes. The volunteers were all men, aged between 21–33 years old. Informed consent was obtained from all the participants as described under the ethics considerations section. In each test, we worked with two volunteers, each working for a maximum of six hours a night.

### Mosquito attractants tested

#### BG-Lure

The BG-Lure (Biogents HmGb, Regensburg, Germany) consists of a mixture of ammonia, L-lactic acid and caproic acid in undeclared proprietary concentrations. It was used in the second experiment to identify a comparatively effective lure for field use when sampling mosquitoes with a BGM trap. This attractant was purchased directly from the manufacturer.

#### Mbita-5 Lure (MB5)

The MB5 was originally tested in western Kenya and consists of ammonia (2.5%), lactic acid (85%), tetradecanoic acid (0.00025%) and 3-methyl-1-butanol (0.000001%) [27]. This attractant was also supplied by the manufacturer Biogents Company and was used in the second experiment.

#### Ifakara blend (IB)

The Ifakara blend was originally developed and tested at Ifakara Health Institute in Tanzania and has been widely used for trapping malaria mosquitoes because of its ability to attract representative proportions of different mosquito species, similar to those that are typically attracted by humans [28]. This synthetic blend consists in a mixture of ammonia (2.5%), L-lactic acid (85%), propionic acid (0.1%), butanoic acid (1%), pentanoic acid (0.01%), 3-methylbutanoic acid (0.001%), heptanoic acid (0.01%), octanoic acid (0.01%) and tetradecanoic acid (0.01%) [28]. Unlike the BG-Lure and MB5, which were used only in the second experiment, this attractant was used in all the experiments in this study. The IB was also supplied by Biogents.

#### Carbon dioxide gas (CO_2_)

In all the experiments, the lures were supplemented with CO_2_ gas, produced from yeast-molasses fermentation, to activate mosquitoes and synergize the attractive effects [29, 30]. In addition, in the experiments where the lures were compared, we also had a separate configuration where CO_2_ was the only bait in the BGM trap. Thus, we were able to separate effects of the CO_2_ gas itself. The CO_2_ was prepared using 500g molasses and 35g yeast mixed in 2 litres of water [31]. The mix was placed into a 5L plastic container and was delivered through a 60cm long silicon tubing (0.5cm diameter).

### Odour-dispensing methods

Microcapsules encased in a plastic cartridge (here referred to as BG-Cartridge and also supplied by Biogents) were used as dispensers for all the synthetic attractants in the experiments. However, for the experiment where odour-dispensing devices were tested, two other dispensers were added. The first was a batch of nylon strips, where each strip (measuring 26.5 x 1cm) was soaked in a solution of a different constituent of the Ifakara blend as originally described by Okumu *et al*. [32]. The second dispensing device added was the BG-Sachet (also supplied by Biogents), which consisted of the microcapsules of the IB odorant constituents encased inside a thin plastic low-density polyethylene sachet.

### Study procedures

A series of experiments were conducted nightly between 06.00 P.M. and 07.00 A.M. in rural villages in south-eastern Tanzania to address the following three key questions: a) how efficacious is the BGM compared to BGS and HLC for sampling free-flying mosquitoes; b) which of the four mosquito attractants, i.e., BG-Lure + CO_2_, MB5 + CO_2_, IB+ CO_2_ or CO_2_ alone, is the most efficacious for baiting BGM; and c) which of the three dispensing methods, i.e., the nylon strips, the BG-cartridges or the BG-sachets, is the most efficacious for dispensing the IB in the BGM traps. All the experiments were conducted outdoors during the rainy season between the months of April and May of 2016.

#### Experiment 1: Comparison of sampling methods for field collection of mosquitoes

To compare sampling methods for catching African malaria mosquitoes, three methods were selected and evaluated in the field. These included BGM, BGS and HLC. The methods were comparatively evaluated using a 3 x 3 Latin square experiment replicated four times, over 12 nights, and repeated in three different villages. However, in Minepa, additional collections were done to improve sampling effort, so that there were a total of 72 trap nights in that village, compared to 12 trap nights in each of the other two villages. In total, there were 96 trap nights for each of the sampling methods. The BGM and BGS traps were baited with the synthetic mosquito lure, Ifakara blend (IB) [28] supplemented with CO_2_ as described above and were set with the opening at approximately 40 cm above the ground level (Fig 2). The HLC was conducted outdoors by two volunteers, the first volunteer working from 06.00 P.M. until midnight, and the second volunteer working between midnight and 07.00 A.M. The traps were thoroughly cleaned every morning after the end of the nightly experiments.

To assess whether the traps were capturing mosquitoes of similar physiological state as those that naturally attempt to bite humans (represented by HLC), approximately 10% of the female *An*. *gambiae* and *An*. *funestus* collected by each method were dissected under a stereo light microscope. The dissected ovaries observed under 10X magnification under a compound microscope to determine the parity status (presence of stretched, i.e., parous or coiled tracheolar skeins, i.e., nulliparous) [33].

#### Experiment 2: Comparison of attractants used for baiting the BG Malaria traps

To identify the most effective mosquito lure that can be used with the BGM trap for sampling host-seeking *Anopheles* mosquitoes, a 5 x 5 Latin square experiment was conducted. Five BGM traps, placed at five different locations, of which four were baited with either of the four candidate lures, and a fifth BGM was un-baited and used as control. The five different treatments compared in this experiment were a) IB supplemented with CO_2_; b) BG-Lure supplemented with CO_2_; c) MB5 supplemented with CO_2_; d) CO_2_ alone; and e) Control (an unbaited trap). The treatments were randomly rotated across all the five locations nightly. The traps were thoroughly cleaned every morning after the end of the nightly experiments. The experiment was replicated four times, amounting to 20 sampling nights for each treatment, during which each bait type had been to each of the locations four times.

#### Experiment 3: Comparison of the odour-dispensing devices used in the BG Malaria trap

Here, methods dispensing the synthetic mosquito attractants were evaluated to determine which was the most efficacious for use in the BGM. Only the IB candidate attractant was used. We compared nightly mosquito catches in BGM traps baited with the IB [28], but dispensed from either: a) nylon strips [32], b) BG-Sachet or c) BG-Cartridge. A non-baited trap was used as control, so that there were four traps being evaluated. Each night, the traps were placed at four different locations, and the position of the dispensers rotated nightly. The traps were thoroughly cleaned every morning after the end of the nightly experiments. The devices were rotated in four positions in a 4 x 4 Latin square design with 5 replicates, totalling 20 sampling nights for each device, during which each dispenser had been to each of the locations five times.

### Processing of samples

Every morning, the mosquitoes collected during the experiments were transported to the Ifakara Health Institute Vector Laboratory (the VectorSphere) for sorting, identification and counting. Morphological classification was used to group the adults as members of the *An*. *gambiae* complex, *An*. *funestus* group, other *Anopheles* species, or culicines. A sub-sample of the *An*. *gambiae s*.*l*. and *An*. *funestus* mosquitoes of the different species were kept in micro-centrifuge tubes with silica gel for further analysis by Polymerase Chain Reaction (PCR) to identify sibling species.

### Data analysis

The analysis was done using R software version 3.3.2 [34]. In all experiments, Generalized Linear Mixed Effects Statistical Models (GLMMs) in the *lme4* package were used to estimate the number of female mosquitoes of each taxon captured as a function of the different sampling methods, lures or dispensing devices. To account for the over-dispersion in the field data, number of mosquitoes captured (i.e., the mosquito count data of each taxon) were modeled following a negative binomial distribution with log link function [35]. In the first experiment, the main fixed effects were trap type. However, in the second experiment (testing the effect of different lures on the number of mosquito captured), the main fixed effect was type of lure, and in the last experiment (testing the effect of different odour dispensing mechanism), the main fixed effect was type of dispensing devices. To account for the variation in temperatures, winds and any other confounding factors during the study period, the experiment date and trap locations were treated as random factors for each analysis in respective experiments. Relative rates (RR) and 95% Confidence Intervals (CI) were used to estimate the relative influence of each fixed effect. In the parity status experiment, GLMM with binomial likelihood for proportion data in the *lme4* package were used [35]. Proportions of parous mosquitoes were modeled as a function of trap type, while experimental date and trap location was treated as random factors. The estimates of all experiments were considered statistically significantly different if p < 0.05. Additionally, pairwise comparison tests were done using Tukey’s honest significance difference post-hoc test (Tukey’s HSD) to assess differences between individual groups.

### Ethical considerations

Volunteers participating in the study were adequately informed of the study objectives, potential benefits and potential risks, after which written informed consent was obtained. Adequate training on experimental procedures was given to the volunteers. All volunteers participating in mosquito experiments had access to weekly diagnosis of malaria and treatment if they became unwell, but no volunteer in this experiment became unwell. Ethical approval for the study was obtained from Ifakara Health Institute IRB (IHI/IRB/No: 34–2014) and Medical Research Coordination Committee of the National Institute of Medical Research (Certificate No. NIMR/HQ/R.8a/Vol.IX/1903).

## Results

### Efficacy of BGM relative to other mosquito sampling methods: mosquito counts and vector species diversities

During the study period, a total of 31,051 mosquitoes were captured between all the sampling methods tested, representing five genera comprising *Anopheles*, *Culex*, *Mansonia*, *Aedes* and *Coquillettidia* (Table 1). As malaria vectors were the prime focus of interest in this study, further analyses was done on *An*. *gambiae s*.*l*. and *An*. *funestus* mosquitoes.

**Table 1:** Mosquito catches, grouped by taxa, with median number and Interquartile range (IQR) of mosquitoes caught per night by different sampling methods in the study.

Species	Human Landing Catch			BG-Malaria Trap			BG-Sentinel Trap		
	Median catch/night (IQR)	Total catch	Proportion	Median catch/night (IQR)	Total catch	Proportion	Median catch/night (IQR)	Total catch	Proportion
*Anopheles gambiae* s.l.	39 (15.5–9.0)	6369	56.0%	5.5 (1.0–15.25)	1083	10.2%	1.5 (0.0–6.25)	691	7.6%
*Anophels funestus*	0 (0–1)	71	0.6%	0 (0–1)	83	0.8%	0 (0–0)	52	0.6%
*Anopheles coustani*	1 (0–2)	354	3.1%	0 (0–1)	92	0.9%	0 (0–0)	64	0.7%
*Anopheles pharoensis*	0 (0–0)	58	0.5%	0 (0–0)	1	0.0%	0 (0–0)	3	0.0%
*Anopheles ziemani*	0 (0–0)	11	0.1%	0 (0–0)	1	0.0%	0 (0–0)	15	0.2%
*Anopheles squamosus*	0 (0–0)	33	0.3%	0 (0–0)	9	0.1%	0 (0–0)	6	0.1%
*Culex* spp.	32 (15.0–54.25)	3757	33.1%	66.5 (22.5–142.0)	8627	81.6%	59 (23.5–103.0)	7821	85.9%
*Mansonia* spp.	2 (0–10)	677	6.0%	2 (0–5.25)	617	5.8%	1 (0–5)	382	4.2%
*Aedes* spp.	0 (0–0)	25	0.2%	0 (0–0)	38	0.4%	0 (0–0)	21	0.2%
*Coquillettidia* spp.	0 (0–0)	11	0.1%	0 (0–0)	25	0.2%	0 (0–0)	54	0.6%
**Total**		**11366**	**100**%		**10576**	**100**%		**9109**	**100**%

The number of *An*. *gambiae s*.*l*. caught was influenced significantly by type of sampling method used (p < 0.001) (Table 2). Nevertheless, BGM and BGS captured similar numbers of *An*. *funestus* compared to HLC (RR = 1.2, 95%CI: (0.62–2.32), p = 0.587) and (RR = 0.71 (0.36–1.39, p = 0.314), respectively.

**Table 2:** Estimation of sampling efficiency of the different sampling methods used (per night) relative to the human landing catch.

*Anopheles gambiae s*.*l*.			*Anopheles funestus*		
Trap	Mean Catch ± SE	OR (95%CI)	p value	Mean Catch ± SE	OR (95%CI)	p value
Human Landing Catch	66.34 ± 6.5	1	N/A	0.74 ± 0.2	1	N/A
BG-Malaria	11.28 ± 1.5	0.16 (0.11–0.24)	<0.001	0.86 ± 0.2	1.20 (0.62–2.32)	0.587
BG-Sentinel	7.20 ± 2	0.08 (0.05–0.12)	<0.001	0.54 ± 0.1	0.71 (0.36–1.39)	0.314

### Efficacy of BGM relative to other mosquito sampling methods: Parity rates

**Fig 3:**
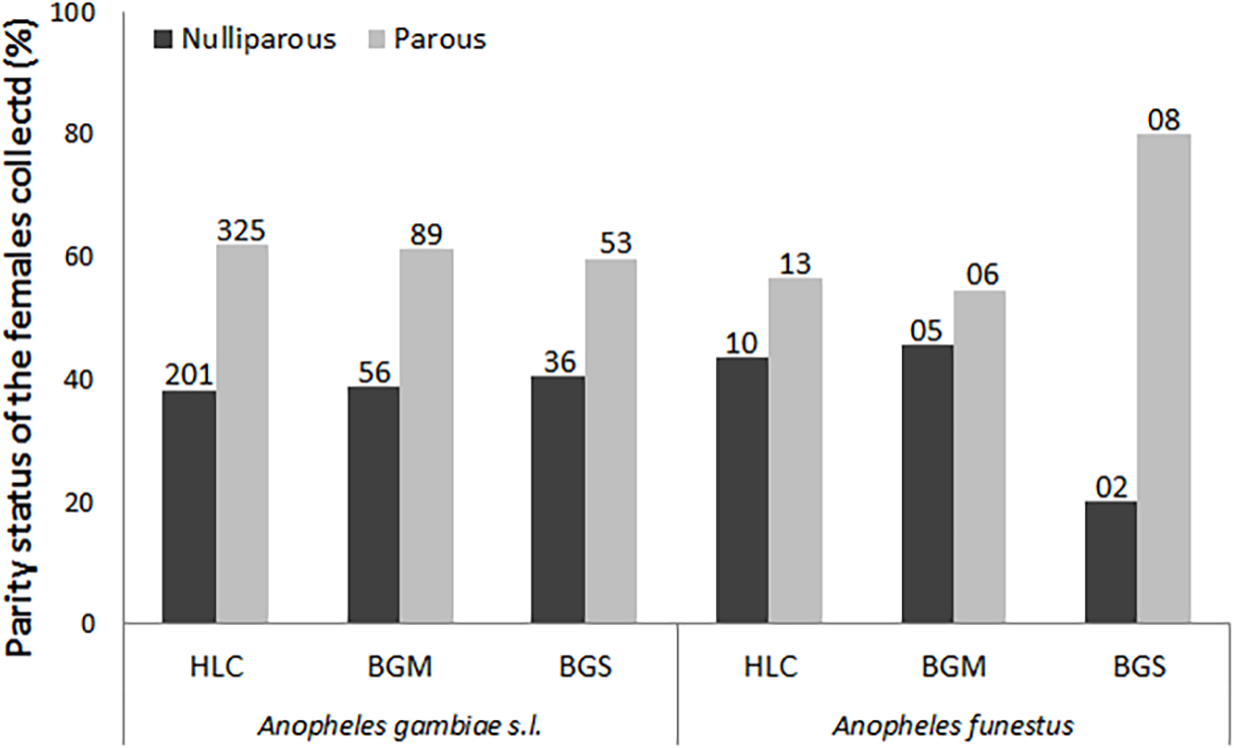
Percentage of parous mosquitoes observed among collections using human-landing catches (HLC), BG-Malaria (BGM) and BG-Sentinel (BGS) traps. The number of mosquitoes dissected per method is included at the top of the bars.

Approximately 10% (804/8349) of the *An*. *gambiae s*.*l*. and *An*. *funestus* females collected in these experiments were dissected to determine their parity status. Proportions of parous *An*. *gambiae s*.*l*. and *An*. *funestus* exceeded 50% with all methods, but no significant difference in parity rates was observed between the methods (Fig 3). The highest parity rate was observed in BGS, where 80% of the *An*. *funestus* dissected were parous, but the overall number of *An*. *funestus* dissected was limited compared *An*. *gambiae s*.*l*.

### Efficacy of mosquito lures used for baiting the BGM

The total numbers of mosquitoes collected in the second experiment, where different synthetic attractants were tested with the BGM trap, are shown in Table 3. As expected, the lowest number of *An*. *gambiae s*.*l*. was collected by the unbaited trap, which was significantly lower than any of the other traps (p < 0.001) (Table 4). Pair-wise comparison test using Tukey’s HSD showed that there is significant difference between the pairs formed by each treatment and control (CO_2_ –control [z = 5.04, p < 0.001], BG-Lure–control [z = 4.82, p < 0.001], MB5 –control [z = 5.05, p < 0.001], IB–control [z = 5.19, p < 0.001]). The rest of the pairs were not significantly different from one another.

**Table 3:** Mosquito catches, grouped by taxa, caught by BG-Malaria traps baited with different lures (CO_2_ gas, BG-Lure, Mbita-5 Blend, Ifakara blend or no bait, i.e., control).

Species	Control		CO_2_		BG-Lure		Mbita-5 Blend		Ifakara Blend	
	Median catch/night (IQR)	Total catch (Proportion)	Median catch/night (IQR)	Total catch (Proportion)	Median catch/night (IQR)	Total catch (Proportion)	Median catch/night (IQR)	Total catch (Proportion)	Median catch/night (IQR)	Total catch (Proportion)
*Anopheles gambiae s*.*l*.	0 (0–0)	4 (20%)	1 (0–3.5)	158 (19.7%)	1 (0–0)	89 (16.2%)	2 (0.75–6.25)	126 (19.4%)	2 (0–5)	115 (12.7%)
*Anopheles funestus*	0 (0–0)	0 (0%)	0 (0–0)	0 (0%)	0 (0–0)	2 (0.4%)	0 (0–0)	4 (0.6%)	0 (0–0)	4 (0.4%)
*Anopheles coustani*	0 (0–0)	1 (5%)	0 (0–0.25)	11 (1.4%)	0 (0–0)	22 (4%)	0 (0–1)	13 (2%)	0 (0–1.25)	114 (12.5%)
*Anopheles pharoensis*	0 (0–0)	1 (5%)	0 (0–0)	2 (0.3%)	0 (0–0)	2 (0.4%)	0 (0–0)	0 (0%)	0 (0–0)	12 (1.3%)
*Anopheles ziemani*	0 (0–0)	0 (0%)	0 (0–0)	0 (0%)	0 (0–0)	1 (0.2%)	0 (0–0)	0 (0%)	0 (0–0)	0 (0%)
*Anopheles squamosus*	0 (0–0)	0 (0%)	0 (0–0)	1 (0.1%)	0 (0–0)	0 (0%)	0 (0–0)	0 (0%)	0 (0–0)	0 (0%)
*Culex* spp.	0 (0–0.25)	11 (55%)	11 (3.75–40.75)	519 (64.7%)	10 (3–23.25)	374 (68.1%)	13 (3.25–35.75)	444 (68.3%)	9 (4.75–47.25)	551 (60.7%)
*Mansonia* spp.	0 (0–0)	3 (15%)	1 (0–4)	109 (13.6%)	0 (0–0)	54 (9.8%)	1 (0–5)	59 (9.1%)	1 (0–5)	103 (11.3%)
*Aedes* spp.	0 (0–0)	0 (0%)	0 (0–0)	1 (0.1%)	0 (0–0)	3 (0.5%)	0 (0–0)	3 (0.5%)	0 (0–0)	4 (0.4%)
*Coquillettidia* spp.	0 (0–0)	0 (0%)	0 (0–0)	1 (0.1%)	0 (0–0)	2 (0.4%)	0 (0–0)	1 (0.1%)	0 (0–0)	6 (0.7%)
**Total**		**20**		**802**		**549**		**650**		**909**

**Table 4:** Estimation of sampling efficiency of BG-Malaria traps baited with different lures (per night) relative to control (unbaited trap)*.

Lure	Mean Catch ± SE	OR (95%CI)	p value
Control	0.20 ± 0.15	1	N/A
CO_2_	7.90 ± 3.83	29.45 (7.90–109.72)	<0.001
BG-Lure	4.45 ± 1.66	26.16 (6.93–98.70)	<0.001
Mbita-5	6.30 ± 2.45	30.03 (8.69–112.3)	<0.001
Ifakara Blend	5.75 ± 2.43	32.23 (8.69–119.56)	<0.001

*Analysis was only done for *Anopheles gambiae s*.*l*., *Anopheles funestus* were too few to perform a robust analysis

### Efficacy of different odour-dispensing devices used in the BGM

The total number of mosquitoes collected in the third experiment, where different devices were tested for dispensing the IB in the BGM trap, is shown in Table 5. A total of 10,448 mosquitoes were collected, distributed in five genera. The proportional composition of mosquitoes of different species making up the total catch remained consistent with the first and second experiments. Here, 83.5% of all the mosquitoes caught were *Culex* spp., 10.5% were *An*. *gambiae s*.*l*., and 3.8% were *Mansonia* spp. (Table 5). All the odour-dispensing devices performed significantly better than control for capturing *An*. *gambiae s*.*l*. mosquitoes, with BG-Sachet being the best odour-dispensing system [RR = 70.71 (23.59–212.00)), p < 0.001], followed by BG-Cartridge [RR = 48.46 (16.15–145.44), p < 0.001] and nylon strips [RR = 18.67 (6.08–57.35), p < 0.001]. The Tukey’s pair-wise comparison showed a difference between BG-Sachet and nylon strips (z = 2.78, p < 0.05), but none of the other pairs were significantly different (Table 6).

**Table 5:** Mosquitoes, grouped by taxa, caught by BG-Malaria traps baited with Ifakara blend released by different odour-dispensing devices (BG-Cartridge, BG-Sachet, Nylon strips or no bait, i.e., control).

Species	Control		BG-Cartridge		BG-Sachet		Nylon Strips	
	Median catch/night (IQR)	Total catch (Proportion)	Median catch/night (IQR)	Total catch (Proportion)	Median catch/night (IQR)	Total catch (Proportion)	Median catch/night (IQR)	Total catch (Proportion)
*Anopheles gambiae s*.*l*.	0 (0–1)	8 (4.3%)	7.5 (1.75–26.25)	388 (11.7%)	14.5 (2.75–42.50)	555 (15.7%)	4.5 (0–10)	145 (4.2%)
*Anopheles funestus*	0 (0–0)	0 (0%)	0 (0–0)	5 (0.2%)	0 (0–2.5)	33 (1%)	0 (0–0)	17 (0.5%)
*Anopheles coustani*	0 (0–0)	0 (0%)	0 (0–1)	29 (0.9%)	0 (0–1)	47 (1.2%)	0 (0–1)	17 (0.5%)
*Anopheles pharoensis*	0 (0–0)	0 (0%)	0 (0–0)	7 (0.2%)	0 (0–0)	5 (0.2%)	0 (0–0)	0 (0%)
*Anopheles ziemani*	0 (0–0)	0 (0%)	0 (0–0)	0 (0%)	0 (0–0)	0 (0%)	0 (0–0)	1 (0.03%)
*Anopheles squamosus*	0 (0–0)	1 (0.5%)	0 (0–0)	0 (0%)	0 (0–0)	3 (0.1%)	0 (0–0)	20 (0.6%)
*Culex* spp.	5 (0–10.5)	173 (94.1%)	128.5 (48.0–177.25)	2641 (79.9%)	133 (52.5–180.25)	2770 (78.6%)	131 (74.25–227.0)	3137 (91.8%)
*Mansonia* spp.	0 (0–0)	2 (1.1%)	4 (0–8.25)	222 (6.7%)	4 (0–7.25)	108 (3.1%)	0.5 (0–7)	69 (2%)
*Aedes* spp.	0 (0–0)	0 (0%)	0 (0–0.25)	6 (0.2%)	0 (0–0)	1 (0.03%)	0 (0–0.25)	6 (0.2%)
*Coquillettidia* spp.	0 (0–0)	0 (0%)	0 (0–0)	5 (0.2%)	0 (0–0)	4 (0.1%)	0 (0–0)	7 (0.2%)
**Total**		**184**		**3303**		**3526**		**3419**

**Table 6:** Pair-wise *post hoc* comparison using Tukey’s honestly significance tests (Tukey’s HSD) showing similarities and differences between number of *Anopheles gambiae* caught by BG-Malaria traps baited with Ifakara blend released by different odour-dispensing devices.

Treatment pairs*	*z* value	*p* value
BG-Sachet/BG-Cartridge	0.85	0.827
BG-Sachet/Nylon strips	2.93	<0.05
BG-Sachet/Control	7.60	<0.001
BG-Cartridge/Nylon strips	2.11	0.147
BG-Cartridge/Control	6.92	<0.001
Nylon strips/Control	5.11	<0.001

*The pairs are listed according to the effectiveness of the odour-dispensing devices.

### Identification of sibling species of *Anopheles gambiae s*.*l*. and *Anopheles funestus* group

A sub-sample of the mosquitoes visually identified as *An*. *gambiae s*.*l*. and belonging to the *An*. *funestus* group were tested using PCR to identify their species. Of the 442 *An*. *gambiae s*.*l*. tested, DNA from 419 (94.8%) were successfully amplified, and all these mosquitoes were identified as *An*. *arabiensis* (100%). The amplification rate for the *An*. *funestus* group was 94.5% (52/55), of which 61.5% were *An*. *funestus s*.*s*. Giles, 32.7% were *Anopheles leesoni* and 5.8% were *An*. *rivulorum*.

## Discussion

This current study is the first field validation of the BGM in Africa and provides initial evidence of its performance against wild free-flying mosquitoes. To validate the potential of BGM for surveillance of disease-transmitting mosquitoes, particularly malaria vectors, we designed field experiments to compare it with existing standard sampling methods and commercially available traps, HLC and BGS. We also tested different attractants and odour-dispensing devices to use with the BGM. The BGS trap originally was designed for capturing *Aedes aegypti* [16], the vector of dengue fever, zika virus and chikungunya, but it has also been shown to be an effective sampling method for anophelines [17, 36–38]. In previous studies where BGS was compared to Suna Trap, CDC light trap and Mosquito Magnet X (MM-X) trap, the BGS trap caught higher numbers of *An*. *gambiae s*.*l*. than these other traps [39]. Schmied *et al*. [17] also demonstrated a higher catching efficiency for *An*. *gambiae s*.*s*. when compared to the MM-X trap in a semi-field setting.

Earlier studies in Brazil demonstrated that a minor adaptation of the BGS, involving using it upside down and using a black and white colour pattern, resulted in significantly higher catches [20]. These results recently have been validated in a semi-field assessments in Tanzania where the adaptation (i.e., the BGM) was evaluated against BGS [22]. In those semi-field tests, the BGM proved to be more efficient in catching the malaria vector *An*. *arabiensis* than BGS [22]. In this study, the superior performance of the BGM trap over the BGS trap in terms of nightly mosquito catches was also observed in the field. The BGM trap caught almost double the number of *An*. *gambiae s*.*l*. and also a higher number of mosquitoes from the other genera (*Culex*, *Mansonia* and *Aedes*). These results suggest that the BGM trap could be used for monitoring various mosquito vectors of human pathogens, including the malaria vectors. One clear disadvantage, however, is that though HLC caught mostly *An*. *gambiae* (constituting 56% of total catches), both BGM and BGS predominantly caught *Culex* mosquitoes (constituting >80% of total catches), with *An*. *gambiae* being only about 10% of overall catches. This is an important parameter to assess representativeness of the BGM and BGS traps when used for surveillance of disease-transmitting mosquitoes.

Several attempts have been made to identify improved surveillance options for host-seeking mosquitoes, but a clear replacement for human landing catches remains elusive. Although there is no trapping device available that can successfully compete with HLC, the BGM trap previously has captured similar numbers of *An*. *darlingi*, the main vector of malaria in Brazil [20]. In the present study, the BGM trap captured more *An*. *funestus*, a species that is also a very anthropophilic mosquito, and it is a competent malaria vector [40], just like *An*. *gambiae s*.*s*., which is considered the most important vector species in Africa [41]. However, the number of *An*. *funestus* in this study was very low, so this specific outcome cannot be considered conclusive.

Due to large-scale use of vector control interventions such as LLINs and IRS, *An*. *gambiae s*.*s*. populations have decreased significantly, and there has also been a shift in the composition of malaria vector species in many places. Since 2000, particularly in the period following massive scale-up of LLINs, many sites in East Africa have reported that *An*. *arabiensis* is becoming the most abundant malaria vector [5, 42, 43], although *An*. *funestus*, which occurs in far lower densities than the former, is clearly the more dominant vector, transmitting nearly nine out of every ten new malaria cases in rural south eastern Tanzania [23]. Moreover, while *An*. *arabiensis* presents an opportunistic behaviour, feeding both on humans and animals [44, 45], *An*. *funestus* preferentially feeds on humans. Its persistence as well the high sporozoite prevalence rates it harbours, consequently leads to far greater increases in entomological inoculation rates (EIR). This has been reported by Lwetoijera *et al*. [43] and by Kaindoa *et al*. [26], both of whom showed that *An*. *funestus* is a highly efficient malaria vector in the same area in rural Tanzania, where the current study was conducted. Overall, monitoring these vector dynamics require a new set of sampling tools that are easy to use, low cost and standardisable across settings, but also those that can measure the core behaviour of the vectors such as their biting behaviours [46].

In recent years, certain major breakthroughs have been made in the search for better surveillance methods. Examples include development of highly effective synthetic lures similar to natural host odours [27, 28] and recent development of the mosquito electrocuting trap [13, 14] and the MosqTent [47], which compared sufficiently with human landing catches when field-tested for sampling malaria vectors. Although comparable to HLC, these mentioned traps are based on the same system, regardless protection, the presence of a volunteer is still required, which can be susceptible to an error bias due to the difference in attractiveness among humans, thus hindering the standardization of such methods. Therefore, the use of traps with synthetic attractants, which mimic human odours without exposing them to risks, can be an alternative to these methods. In this context, the combination of odour-baited devices with electric grids [15], and the recent adaptation of BGS to form BGM, which also proved very efficient when compared against human landing catches for field-collections of *An*. *darlingi* [20], are good examples. Our findings offer new evidence for the performance and potential of the BGM in African settings, where despite having lower catches than HLC, it could be an improvement over the current alternatives such as BGS.

According to Mboera [48], a trap can be considered effective for capturing anthropophilic anophelines if its performance is comparable to that of HLC, not only in number of specimens captured, but also in the parity ratio of the females. In this current study, dissections of 10% of all *An*. *gambiae s*.*l*. and *An*. *funestus* specimens collected showed no difference in the proportions of parous females captured by BGM and HLC, corroborating the findings of Gama *et al*. in Brazil [20], who reported a similar physiological conditions in the sampled specimens. Besides demonstrating higher likelihood of being infected, monitoring parous females could be effective as a means of assessing the effectiveness of control interventions. Although not catching more mosquitoes than human attraction, the BGM trap was sufficiently comparable to HLC on the basis of the parity rates in both the two vector species assessed. In these experiments, both BGM and BGS also were capturing malaria vectors of same physiological state as human landing catches. All mosquitoes collected were unfed, and when dissected, we observed that the proportion of parous mosquitoes was consistently above 50% across all trap types, and that there was no difference between the traps.

The preference for feeding on humans displayed by anthropophilic mosquitoes has led to the development of odour blends for attracting these mosquitoes through a process of analysing the attractiveness of volatiles produced by humans [49–52]. The synthetic human odour developed at Ifakara Health Institute (Ifakara Blend) is an example of this type of blend, and it was demonstrated to be more attractive than humans at medium to long range [28]. In our findings, this blend was also highly attractive, although there were no statistical differences with the other lures tested for capturing *An*. *gambiae s*.*l*. All the synthetic blends tested in several studies were added with CO_2_, a routine procedure to enhance mosquito responses [28, 36, 38, 53, 54]. Nevertheless, here the traps baited with CO_2_ alone attracted similar numbers of *An*. *gambiae s*.*l*. as traps baited with the synthetic attractants evaluated. Since the role of the CO_2_ in the attraction of host-seeking anophelines is well known [29, 55, 56], different sources, other than pressurized cylinders and dry ice, have been evaluated to use in traps to reduce the costs [31, 57, 58]. Yeast-fermented molasses, which has been demonstrated previously to be an effective and low-cost alternative source of CO_2_ for odour-baited trapping systems of mosquitoes [31], was used in our experiments. Our results confirm these previous findings, showing that a trap baited with CO_2_ originated from this mixture together with a synthetic lure can be effective.

In addition to having a good attractant, the success of odour-baited tools also is influenced by the selected odour-dispensing devices. In our experiments, the highest catches of *An*. *gambiae s*.*l*. were associated with the IB-treated microcapsules from Biogents Company encased in a plastic sachet. In other studies, nylon strips were the most effective matrix for dispensing synthetic attractants [32, 59]. However, the results demonstrated in this study shown that polymer materials such as the microcapsules on the sachets and plastic cartridge perform better than nylon strips for monitoring malaria mosquitoes. The same result was also reported in our semi-field study [22], where polymer materials were more effective for sampling *An*. *arabiensis* and also in a recent study conducted in Porto Velho, Brazil, with other polymer-based odour-dispensing devices (Batista *et al*., unpublished). Elsewhere, Mweresa *et al*. [60] showed that alternative textile materials such as cotton and polyester could be more effective than nylon as a sustainable dispenser for synthetic attractants of host-seeking *Anopheles* mosquitoes. However, such devices do not allow control odour dispensation and are therefore short-lasting. Ideally, the odour dispensers should provide a constant and long-lasting efficacy in field sampling.

In our results, this trap is an effective tool for sampling mosquitoes. Nevertheless, there is need for a more refined attractant to yield representative vector samples as HLC. In our view, this remains priority if the new trap designs such as BGM and BGS are to be implemented widely. One other gap is that we did not test the devices for sampling indoor-biting vectors. Even though the residual malaria vectors increasingly bite outdoors, it is essential that a surveillance tool works properly in both indoor and outdoor settings. Additional studies in field settings are therefore recommended, both for validating and improving functionality of the BGM trap, but also for assessing its potential when used in intervention programs such as mass trapping [61] or in push-pull systems [62, 63].

## Conclusion

We have described the BGM trap as a potential tool for field-sampling malaria vectors in Africa. The performance of this trap is vastly improved when used with an effective attractant, such as the Ifakara Blend, dispensed using an effective device such as polymer materials encased in the BG-Sachet. The physiological states of malaria vectors caught in BGM and BGS were similar to those naturally attempting to bite humans. However, both BGM and BGS predominantly caught more culicines than *Anopheles*, compared to HLC, which caught mostly *An*. *gambiae s*.*l*. Therefore, even though the trap have potential for field-sampling of African malaria vectors, the lack of representativeness of mosquito species diversity may limit its application as alternative for field-sampling of *Anopheles* mosquitoes.

## References

[1] WHO. World Malaria Report 2017 Geneva: World Health Organization 2017.

[2] Bhatt S, Weiss D, Cameron E, Bisanzio D, Mappin B, Dalrymple U, et al The effect of malaria control on *Plasmodium falciparum* in Africa between 2000 and 2015. Nature. 2015;526(7572):207–11. 10.1038/nature15535 26375008 PMC4820050

[3] WHO. A global brief on vector-borne diseases Geneva: World Health Organization 2014.

[4] Mayer SV, Tesh RB, Vasilakis N. The emergence of arthropod-borne viral diseases: A global prospective on dengue, chikungunya and zika fevers. Acta tropica. 2017;166:155–63. 10.1016/j.actatropica.2016.11.020 27876643 PMC5203945

[5] Killeen GF, Seyoum A, Sikaala C, Zomboko AS, Gimnig JE, Govella NJ, et al Eliminating malaria vectors. Parasites & Vectors. 2013;6:172. 23758937 10.1186/1756-3305-6-172 PMC3685528

[6] Russell TL, Govella NJ, Azizi S, Drakeley CJ, Kachur SP, Killeen GF. Increased proportions of outdoor feeding among residual malaria vector populations following increased use of insecticide-treated nets in rural Tanzania. Malaria Journal. 2011;10(80):80. 21477321 10.1186/1475-2875-10-80 PMC3084176

[7] Bayoh MN, Mathias DK, Odiere MR, Mutuku FM, Kamau L, Gimnig JE, et al *Anopheles gambiae*: historical population decline associated with regional distribution of insecticide-treated bed nets in western Nyanza Province, Kenya. Malaria Journal. 2010;9(1):62. 20187956 10.1186/1475-2875-9-62 PMC2838909

[8] Mwangangi JM, Mbogo CM, Orindi BO, Muturi EJ, Midega JT, Nzovu J, et al Shifts in malaria vector species composition and transmission dynamics along the Kenyan coast over the past 20 years. Malaria Journal. 2013;12(1):1–9. 23297732 10.1186/1475-2875-12-13 PMC3544599

[9] Killeen GF, Marshall JM, Kiware SS, South AB, Tusting LS, Chaki PP, et al Measuring, manipulating and exploiting behaviours of adult mosquitoes to optimise malaria vector control impact. BMJ Global Health. 2017;2(2). 10.1136/bmjgh-2016-000212 PMC5444085 28589023

[10] Moiroux N, Gomez MB, Pennetier C, Elanga E, Djènontin A, Chandre F, et al Changes in *Anopheles funestus* biting behavior following universal coverage of long-lasting insecticidal nets in Benin. Journal of Infectious Diseases. 2012;206(10):1622–9. 10.1093/infdis/jis565 22966127

[11] Zhu L, Müller GC, Marshall JM, Arheart KL, Qualls WA, Hlaing WM, et al Is outdoor vector control needed for malaria elimination? An individual-based modelling study. Malaria Journal. 2017;16(1):266 10.1186/s12936-017-1920-y 28673298 PMC5496196

[12] Lima JBP, Rosa-Freitas MG, Rodovalho CM, Santos F, Lourenço-de-Oliveira R. Is there an efficient trap or collection method for sampling *Anopheles darlingi* and other malaria vectors that can describe the essential parameters affecting transmission dynamics as effectively as human landing catches?-A Review. Memorias do Instituto Oswaldo Cruz. 2014;109(5):685–705. 10.1590/0074-0276140134 25185008 PMC4156462

[13] Maliti DV, Govella NJ, Killeen GF, Mirzai N, Johnson PC, Kreppel K, et al Development and evaluation of mosquito-electrocuting traps as alternatives to the human landing catch technique for sampling host-seeking malaria vectors. Malaria Journal. 2015;14(1):502. 26670881 10.1186/s12936-015-1025-4 PMC4681165

[14] Govella NJ, Maliti DF, Mlwale AT, Masallu JP, Mirzai N, Johnson PC, et al An improved mosquito electrocuting trap that safely reproduces epidemiologically relevant metrics of mosquito human-feeding behaviours as determined by human landing catch. Malaria Journal. 2016;15(1):465. 27618941 10.1186/s12936-016-1513-1 PMC5020444

[15] Matowo NS, Koekemoer LL, Moore SJ, Mmbando AS, Mapua SA, Coetzee M, et al Combining Synthetic Human Odours and Low-Cost Electrocuting Grids to Attract and Kill Outdoor-Biting Mosquitoes: Field and Semi-Field Evaluation of an Improved Mosquito Landing Box. PloS One. 2016;11(1). 10.1371/journal.pone.0145653 PMC4720432 26789733

[16] Kroeckel U, Rose A, Eiras ÁE, Geier M. New tools for surveillance of adult yellow fever mosquitoes: Comparison of trap catches with human landing rates in an urban environment. Journal of the American Mosquito Control Association. 2006;22(2):229–38. 10.2987/8756-971X(2006)22[229:NTFSOA]2.0.CO;2 17019768

[17] Schmied WH, Takken W, Killeen GF, Knols BG, Smallegange RC. Evaluation of two counterflow traps for testing behaviour-mediating compounds for the malaria vector *Anopheles gambiae ss* under semi-field conditions in Tanzania. Malaria Journal. 2008;7(1):230. 18980669 10.1186/1475-2875-7-230 PMC2585591

[18] Pombi M, Guelbeogo WM, Calzetta M, Sagnon NF, Petrarca V, La Gioia V, et al Evaluation of a protocol for remote identification of mosquito vector species reveals BG-Sentinel trap as an efficient tool for *Anopheles gambiae* outdoor collection in Burkina Faso. Malaria Journal. 2015;14(1):161. 25888896 10.1186/s12936-015-0674-7 PMC4406007

[19] Okal MN, Herrera-Varela M, Ouma P, Torto B, Lindsay SW, Lindh JM, et al Analysing chemical attraction of gravid *Anopheles gambiae sensu stricto* with modified BG-Sentinel traps. Parasites & Vectors. 2015;8(1):301. 26036270 10.1186/s13071-015-0916-0 PMC4456765

[20] Gama RA, Silva IMd, Geier M, Eiras ÁE. Development of the BG-Malaria trap as an alternative to human-landing catches for the capture of *Anopheles darlingi*. Memórias do Instituto Oswaldo Cruz. 2013;108(6):763–71. 10.1590/0074-0276108062013013 24037199 PMC3970694

[21] Rodrigues MS, Silva IM, Leal LB, Dos Santos CA Jr, Eiras ÁE. Development of a New Mosquito Retention System for the BG-Malaria Trap To Reduce The Damage To Mosquitoes. Journal of the American Mosquito Control Association. 2014;30(3):184–90. 10.2987/14-6423R.1 25843093

[22] Batista EP, Ngowo HS, Opiyo M, Shubis GK, Meza FC, Okumu FO, et al Semi-field assessment of the BG-Malaria trap for monitoring the African malaria vector, *Anopheles arabiensis*. PLoS One. 2017;12(10):e0186696 10.1371/journal.pone.0186696 29045484 PMC5646867

[23] Kaindoa EW, Mkandawile G, Ligamba G, Kelly-Hope LA, Okumu FO. Correlations between household occupancy and malaria vector biting risk in rural Tanzanian villages: implications for high-resolution spatial targeting of control interventions. Malaria Journal. 2016;15(1):1. 27067147 10.1186/s12936-016-1268-8 PMC4828883

[24] Oketch F, Kotas M, Kihonda J, Killeen G, Moore S. Comparative evaluation of methods used for sampling malaria vectors in the Kilombero Valley, South Eastern Tanzania. The Open Tropical Medicine Journal. 2008;1:51–5.

[25] Okumu FO, Madumla EP, John AN, Lwetoijera DW, Sumaye RD. Attracting, trapping and killing disease-transmitting mosquitoes using odor-baited stations-The Ifakara Odor-Baited Stations. Parasites & Vectors. 2010;3:12. 20193085 10.1186/1756-3305-3-12 PMC2838860

[26] Kaindoa EW, Matowo NS, Ngowo HS, Mkandawile G, Mmbando A, Finda M, et al Interventions that effectively target *Anopheles funestus* mosquitoes could significantly improve control of persistent malaria transmission in south–eastern Tanzania. PLoS One. 2017;12(5):e0177807 10.1371/journal.pone.0177807 28542335 PMC5436825

[27] Mukabana WR, Mweresa CK, Otieno B, Omusula P, Smallegange RC, van Loon JJA, et al A novel synthetic odorant blend for trapping of malaria and other African mosquito species. Journal of Chemical Ecology. 2012;38(3):235–44. 10.1007/s10886-012-0088-8 22426893 PMC3310138

[28] Okumu FO, Killeen GF, Ogoma S, Biswaro L, Smallegange RC, Mbeyela E, et al Development and field evaluation of a synthetic mosquito lure that is more attractive than humans. PloS One. 2010;5(1):e8951 10.1371/journal.pone.0008951 20126628 PMC2812511

[29] Gillies M. The role of carbon dioxide in host-finding by mosquitoes (Diptera: Culicidae): A review. Bulletin of Entomological Research. 1980;70(04):525–32.

[30] Dekker T, Geier M, Cardé RT. Carbon dioxide instantly sensitizes female yellow fever mosquitoes to human skin odours. Journal of Experimental Biology. 2005;208(15):2963–72. 16043601 10.1242/jeb.01736

[31] Mweresa CK, Omusula P, Otieno B, Van Loon JJ, Takken W, Mukabana WR. Molasses as a source of carbon dioxide for attracting the malaria mosquitoes *Anopheles gambiae* and *Anopheles funestus*. Malaria Journal. 2014;13:160 10.1186/1475-2875-13-160 24767543 PMC4020376

[32] Okumu F, Biswaro L, Mbeleyela E, Killeen G, Mukabana R, Moore S. Using nylon strips to dispense mosquito attractants for sampling the malaria vector *Anopheles gambiae ss*. Journal of Medical Entomology. 2010;47(2):274–82. 20380310 10.1603/me09114

[33] WHO. Manual on Pratical Entomology in Malaria—Methods and Techniques Geneva: World Health Organization 1975.

[34] Team RC. R: A language and environment for statistical computing R Foundation for Statistical Computing, Vienna, Austria 2015, URL http://www.R-projectorg. 2016.

[35] Bates D, Mächler M, Bolker B, Walker S. Fitting linear mixed-effects models using lme4. arXiv preprint arXiv:14065823. 2014.

[36] Busula AO, Takken W, Loy DE, Hahn BH, Mukabana WR, Verhulst NO. Mosquito host preferences affect their response to synthetic and natural odour blends. Malaria Journal. 2015;14(1):133. 25889954 10.1186/s12936-015-0635-1 PMC4381365

[37] Gama RA, Silva IMd, Monteiro HAdO, Eiras ÁE. Fauna of Culicidae in rural areas of Porto Velho and the first record of *Mansonia (Mansonia) flaveola* (Coquillet, 1906), for the state of Rondônia, Brazil. Revista da Sociedade Brasileira de Medicina Tropical. 2012;45(1):125–7. 22370843 10.1590/s0037-86822012000100025

[38] Hoel DF, Marika JA, Dunford JC, Irish SR, Geier M, Obermayr U, et al Optimizing Collection of *Anopheles gambiae ss* (Diptera: Culicidae) in Biogents Sentinel Traps. Journal of Medical Entomology. 2014;51(6):1268–75. 10.1603/ME14065 26309317

[39] Verhulst NO, Bakker JW, Hiscox A. Modification of the Suna Trap for Improved Survival and Quality of Mosquitoes in Support of Epidemiological Studies. Journal of the American Mosquito Control Association. 2015;31(3):223–32. 10.2987/moco-31-03-223-232.1 26375903

[40] Mendis C, Jacobsen J, Gamage‐Mendis A, Bule E, Dgedge M, Thompson R, et al *Anopheles arabiensis* and *An*. *funestus* are equally important vectors of malaria in Matola coastal suburb of Maputo, southern Mozambique. Medical and Veterinary Entomology. 2000;14(2):171–80. 10872861 10.1046/j.1365-2915.2000.00228.x

[41] Coetzee M, Craig M, Le Sueur D. Distribution of African malaria mosquitoes belonging to the *Anopheles gambiae* complex. Parasitology Today. 2000;16(2):74–7. 10652493 10.1016/s0169-4758(99)01563-x

[42] Russell TL, Lwetoijera DW, Maliti D, Chipwaza B, Kihonda J, Charlwood JD, et al Research Impact of promoting longer-lasting insecticide treatment of bed nets upon malaria transmission in a rural Tanzanian setting with pre-existing high coverage of untreated nets. Malaria Journal. 2010;9:187 10.1186/1475-2875-9-187 20579399 PMC2902500

[43] Lwetoijera DW, Harris C, Kiware SS, Dongus S, Devine GJ, McCall PJ, et al Increasing role of *Anopheles funestus* and *Anopheles arabiensis* in malaria transmission in the Kilombero Valley, Tanzania. Malaria Journal. 2014;13:331 10.1186/1475-2875-13-331 25150840 PMC4150941

[44] Tirados I CC, Gibson G, Torr S. Blood-feeding behaviour of the malarial mosquito *Anopheles arabiensis*: implications for vector control. Medical and Veterinary Entomology. 2006;20:425–37. 10.1111/j.1365-2915.2006.652.x 17199754

[45] Kelly-Hope LA MF. The mutiplicity of malaria transmission: a review of entomological inoculation rate measurements and methods across sub-saharan Africa. Malaria Journal. 2009;8(19). 10.1186/1475-2875-8-19 PMC2656515 19166589

[46] Govella NJ, Chaki PP, Killeen GF. Entomological surveillance of behavioural resilience and resistance in residual malaria vector populations. Malaria Journal. 2013;12(1):124. 23577656 10.1186/1475-2875-12-124 PMC3637503

[47] Lima JB, Galardo AK, Bastos LS, da Silva Lima AW, Rosa-Freitas MG. MosqTent: An individual portable protective double-chamber mosquito trap for anthropophilic mosquitoes. PLoS neglected tropical diseases. 2017;11(3). 10.1371/journal.pntd.0005245 PMC5344318 28278171

[48] Mboera L. Sampling techniques for adult Afrotropical malaria vectors and their reliability in the estimation of entomological inoculation rate. Tanzania Journal of Health Research. 2006;7(3):117–24. 10.4314/thrb.v7i3.14248 16941936

[49] Qiu Y, Smallegange R, Van Loon J, Ter Braak C, Takken W. Interindividual variation in the attractiveness of human odours to the malaria mosquito *Anopheles gambiae ss*. Medical and Veterinary Entomology. 2006;20(3):280–7. 10.1111/j.1365-2915.2006.00627.x 17044878

[50] Logan JG, Birkett MA, Clark SJ, Powers S, Seal NJ, Wadhams LJ, et al Identification of human-derived volatile chemicals that interfere with attraction of *Aedes aegypti* mosquitoes. Journal of Chemical Ecology. 2008;34(3):308–22. 10.1007/s10886-008-9436-0 18306972

[51] Verhulst NO, Mbadi PA, Kiss GB, Mukabana WR, van Loon JJ, Takken W, et al Improvement of a synthetic lure for *Anopheles gambiae* using compounds produced by human skin microbiota. Malaria Journal. 2011;10(1):1. 21303496 10.1186/1475-2875-10-28 PMC3041721

[52] Mukabana WR, Mweresa CK, Otieno B, Omusula P, Smallegange RC, Van Loon JJ, et al A novel synthetic odorant blend for trapping of malaria and other African mosquito species. Journal of Chemical Ecology. 2012;38(3):235–44. 10.1007/s10886-012-0088-8 22426893 PMC3310138

[53] Menger D, Van Loon J, Takken W. Assessing the efficacy of candidate mosquito repellents against the background of an attractive source that mimics a human host. Medical and Veterinary Entomology. 2014;28(4):407–13. 10.1111/mve.12061 24797537

[54] Mmbando AS, Okumu FO, Mgando JP, Sumaye RD, Matowo NS, Madumla E, et al Effects of a new outdoor mosquito control device, the Mosquito Landing Box, on densities and survival of the malaria vector, *Anopheles arabiensis*, inside controlled semi-field settings. Malaria Journal. 2015;14(1):1. 26645085 10.1186/s12936-015-1013-8 PMC4673850

[55] Dekker T, Takken W. Differential responses of mosquito sibling species *Anopheles arabiensis* and *An*. *quadriannulatus* to carbon dioxide, a man or a calf. Medical and Veterinary Entomology. 1998;12(2):136–40. 9622366 10.1046/j.1365-2915.1998.00073.x

[56] Spitzen J, Smallegange RC, Takken W. Effect of human odours and positioning of CO_2_ release point on trap catches of the malaria mosquito *Anopheles gambiae sensu stricto* in an olfactometer. Physiological Entomology. 2008;33(2):116–22.

[57] Saitoh Y, Hattori J, Chinone S, Nihei N, Tsuda Y, Kurahashi H, et al Yeast-generated CO_2_ as a convenient source of carbon dioxide for adult mosquito sampling. Journal of the American Mosquito Control Association. 2004;20(3):261–4. 15532924

[58] Smallegange RC, Schmied WH, van Roey KJ, Verhulst NO, Spitzen J, Mukabana WR, et al Sugar-fermenting yeast as an organic source of carbon dioxide to attract the malaria mosquito *Anopheles gambiae*. Malaria Journal. 2010;9(1):292. 20973963 10.1186/1475-2875-9-292 PMC2984570

[59] Mukabana WR, Mweresa CK, Omusula P, Orindi BO, Smallegange RC, van Loon JJ, et al Evaluation of low density polyethylene and nylon for delivery of synthetic mosquito attractants. Parasites & Vectors. 2012;5:202. 22992518 10.1186/1756-3305-5-202 PMC3480916

[60] Mweresa CK, Mukabana WR, Omusula P, Otieno B, Gheysens T, Takken W, et al Evaluation of textile substrates for dispensing synthetic attractants for malaria mosquitoes. Parasite & Vectors. 2014;7:376. 10.1186/1756-3305-7-376 PMC4152566 25129505

[61] Degener C, Eiras A, Azara T, Roque R, Rösner S, Codeço C, et al Evaluation of the effectiveness of mass trapping with BG-sentinel traps for dengue vector control: a cluster randomized controlled trial in Manaus, Brazil. Journal of Medical Entomology. 2014;51(2):408–20. 24724291 10.1603/me13107

[62] Obermayr U, Ruther J, Bernier UR, Rose A, Geier M. Evaluation of a push-pull approach for *Aedes aegypti* (L.) using a novel dispensing system for spatial repellents in the laboratory and in a semi-field environment. PLoS One. 2015;10(6):e0129878 10.1371/journal.pone.0129878 26115365 PMC4482593

[63] Salazar FV, Achee NL, Grieco JP, Prabaripai A, Eisen L, Shah P, et al Evaluation of a peridomestic mosquito trap for integration into an *Aedes aegypti* (Diptera: Culicidae) push‐pull control strategy. Journal of Vector Ecology. 2012;37(1):8–19. 10.1111/j.1948-7134.2012.00195.x 22548532

